# Targeted Deletion of the Murine *Lgr4* Gene Decreases Lens Epithelial Cell Resistance to Oxidative Stress and Induces Age-Related Cataract Formation

**DOI:** 10.1371/journal.pone.0119599

**Published:** 2015-03-26

**Authors:** Jun Zhu, Qiang Hou, Xiang Da Dong, Zhenlian Wang, Xiaoyan Chen, Dandan Zheng, Linglin Zhou, Chao He, Mingyao Liu, LiLi Tu, Jia Qu

**Affiliations:** 1 School of Ophthalmology and Optometry, Eye Hospital, Wenzhou Medical University, Wenzhou, Zhejiang, China; 2 State Key Laboratory Cultivation Base and Key Laboratory of Vision Science, Ministry of Health of the People’s Republic of China, Zhejiang Provincial Key Laboratory of Ophthalmology and Optometry, Wenzhou, Zhejiang, China; 3 Department of Surgery, Stamford Hospital—Affiliate of Columbia University, Stamford, Connecticut, United States of America; 4 Institute of Biosciences and Technology and Department of Molecular and Cellular Medicine, Texas A&M University Health Science Center, Houston, Texas, United States of America; University of Colorado Denver School of Medicine, UNITED STATES

## Abstract

Oxidative stress contributes to the formation of cataracts. The leucine rich repeat containing G protein-coupled receptor 4 (LGR4, also known as GPR48), is important in many developmental processes. Since deletion of *Lgr4* has previously been shown to lead to cataract formation in mice, we sought to determine the specific role that *Lgr4* plays in the formation of cataracts. Initially, the lens opacities of *Lgr4^−/−^* mice at different ages without ocular anterior segment dysgenesis (ASD) were evaluated with slit-lamp biomicroscopy. Lenses from both *Lgr4^−/−^* and wild-type mice were subjected to oxidation induced protein denaturation to assess the ability of the lens to withstand oxidation. The expression of antioxidant enzymes was evaluated with real-time quantitative PCR. Phenotypically, *Lgr4^−/−^* mice showed earlier onset of lens opacification and higher incidence of cataract formation compared with wild-type mice of similar age. In addition, *Lgr4^−/−^* mice demonstrated increased sensitivity to environmental oxidative damage, as evidenced by altered protein expression. Real-time quantitative PCR showed that two prominent antioxidant defense enzymes, catalase (CAT) and superoxidase dismutase-1 (SOD1), were significantly decreased in the lens epithelial cells of *Lgr4^−/−^* mice. Our results suggest that the deletion of *Lgr4* can lead to premature cataract formation, as well as progressive deterioration with aging. Oxidative stress and altered expression of several antioxidant defense enzymes contribute to the formation of cataracts.

## Introduction

Age-related cataracts are the most frequent cause of treatable blindness in the world [1]. Cataract formation entails a progressive opacification of the ocular lens, leading to deterioration of the optic image quality formed on the retina, and eventually causing blindness. With increasing life expectancy, the incidence of age-related cataracts will likely increase and produce both a significant medical challenge and socio-economic burden worldwide [2].

Oxidative insult appears to be the most important risk factor for age-related cataract formation [3]. The lens of the vertebrate eye is comprised of a single layer of epithelial cells on its anterior hemispheric surface, with fiber cells that differentiated from epithelial cells making up the bulk of its volume. Most of the metabolic, synthetic, and active transport machineries in the lens are localized to lens epithelial (nucleated) cells. Environmental insults such as oxidative stress and UV radiation are mitigated by epithelial cells through multiple cellular defense mechanisms that begin with altered gene expression [4,5]. The numerous protective mechanisms that have evolved against oxidative stress in the ocular lens make it an excellent model for studying both the biology of aging and the molecular mechanisms associated with oxidative stress.

Several mechanisms have been developed to maintain the redox state of the lens. Aging of the lens is characterized by accumulation of oxidized lens components (especially reactive oxygen species, ROS) and diminished activities of deoxidizing enzymes [6]. Many of the protein and membrane alterations observed in human cataract lenses are due to oxidative origin, and incubation of animal lenses in the presence of hydrogen peroxide (or other oxidants) reproduces many of those changes. Previous works have provided evidence that human lens epithelium is capable of responding to the presence of oxidative stress through the altered expression of numerous genes, including superoxide dismutase, catalase, and glutathione peroxidase [7,8]. A caveat to those findings is that numerous investigations into antioxidants and deoxidizing enzymes are undertaken with the intent of preventing or reversing the damages associated with age-related cataract formation.

*Lgr4* (leucine-rich repeat containing G protein-coupled receptor 4), also known as *Gpr48* (G protein-coupled receptor 48), is one of the genes under active investigation in our laboratory. LGR4 is widely expressed in multiple organs such as intestines, heart, kidneys, cartilage, reproductive tracts and the nervous system, with LGR4 playing important roles in the development of these organs in mice [9–14]. Specifically, we have previously demonstrated that inactivation of *Lgr4* induced an eye open at birth phenotype by reducing epithelial cell proliferation and migration through HB-EGF mediated EGFR activation [15,16]. In another study, we found that deletion of *Lgr4* led to ocular anterior segment dysgenesis (ASD) [17]. Previously, we have described that about 26% of *Lgr4* knockout mice have cataracts, lens fiber disorganization and abnormal protein deposition in lenses [17]. However, its mechanism was unclear.

In this study, we first verified that *Lgr4* null mice had early onset of cataracts, along with description of their phenotype with regards to the cataracts. Subsequently, we determined that *Lgr4* knockout led to age-related cataract formation in mice. We then performed studies to investigate if *Lgr4* deletion attenuated the antioxidative damage ability of lens epithelial cells. Our results will hopefully lead to the identification of novel targets for the prevention and treatment of age-related cataract formation.

## Materials and Methods

### Mice

*Lgr4* ^*+/−*^ mice were generated as previously described [17]. These mice were maintained on a mixed 129 x C57BL/6 background. Heterozygous mice were intercrossed to generate homozygous *Lgr4* ^*−/−*^ mice and wild-type littermate controls. All studies and procedures were approved by the Wenzhou Medical University Animal Care and Use Committee.

Genomic DNA was extracted from mouse tails, and genotypes were determined by PCR analysis using three primers as previously described [17]. Six-week-old *Lgr4* knockout mice that had no ocular ASD were selected and raised for the following experiments. Age-matched wild-type mice were used as negative control. Mice were sacrificed by neck dislocation prior to experiments.

### X-gal staining

To perform X-gal staining, embryonic mice heads or eyes of adult mice were embedded in frozen section medium (Neg-50; Richard-Allen Scientific, Kalamazoo, MI) in preparation for making 10 μm thick frozen sections. The frozen sections were then placed on the slides. The slides were immediately incubated with the β-glycosidase fixative buffer (0.2% glutaraldehyde, 1.5% formaldehyde, 2 mM MgCl_2_, 5 mM EDTA, 0.1 M sodium phosphate buffer, pH 8.0) for 30 minutes and soaked three times for 15 minutes each in the washing buffer (0.1 M sodium phosphate buffer, 2 mM MgCl_2_, 5 mM EDTA, 0.01% sodium deoxycholate, 0.02% Nonidet P40, pH 8.0). Subsequently, the slides were incubated in the staining solution (washing buffer containing 1 mg/mL X-gal, 5 mM K_3_[Fe(CN)_6_], 5 mM K_4_[Fe(CN)_6_]x3H_2_O) until results were optimized. The slides were then counterstained with eosin (Shanghai SSS Reagent Co., Shanghai, China) before they were evaluated and photographed under a microscope (Imager Z1; Carl Zeiss, Oberkochen, Germany).

### Slit-Lamp Biomicroscopy

Selected mice were raised to 70 weeks and the lens changes were observed every week. Mice pupils were dilated with eye drops containing 0.5% tropicamide and 0.5% phenylephrine hydrochloride. Approximately 10 minutes later, the mice were examined with a slit lamp (Photo Slit-Lamp; Haag-Streit AG International, Koeniz, Switzerland). The pictures were analyzed using Eye Cap^TM^ Image Capture Software (Haag-Streit AG International).

### Oxidation Exposure

Fresh clear lenses procured from variably aged mice were pre-incubated in TC-199 medium for 2 hours in a CO_2_ incubator. Lenses were then transferred onto 96-well plates containing 200 μL of fresh TC-199 medium containing 0.1 mM H_2_O_2_ and 2.31 units glucose oxidase (GO) to achieve a final concentration of 0.2 mM H_2_O_2_ and incubated for 24 hours [18]. Lens images were captured from culture plates at 0, 12, and 24 hours after H_2_O_2_ treatment using a dark field microscopy (Olympus BX40; Olympus, Tokyo, Japan).

For the protein denaturation assay, fresh clear lenses from knockout and wild-type mice at different weeks were disrupted in 1,000 μL Tris-HCl buffer (pH 7.4) containing 0.1% glucose, 0.1 mM H_2_O_2_, and 2.31 units GO. The homogenate was centrifuged for 20 minutes at 4°C in a 10,000g microfuge twice. Protein concentrations of the supernatant were determined by BCA assay (Pierce, Rockford, IL) and samples were diluted to 1 mg/ml protein. Samples were then placed in a 37°C water bath for indicated times and measured by a spectrophotometer at 360 nm.

### RNA Isolation and cDNA Synthesis

The lens epithelium was removed by grasping the capsule with fine forceps and carefully peeling it away from the underlying fibrous cell mass as described [19]. RNA isolation was performed using standard protocol. Subsequently, it was treated with DNase I, and reverse-transcribed with RevertAid M-MuLV Reverse Transcriptase using oligo (dT) primers to synthesize the cDNA.

### Determination of mRNA Levels

Gene expression was determined with an ABI Prism 7700 Sequence Detection system (Applied Biosystems, Foster City, CA) using SYBR-green technology. PCR primers were designed using Primer Express 1.5 Software with the manufacturer’s default settings. After PCR amplification, dissociation curves were constructed to confirm formation of the intended PCR products. For quantification, mRNA expression was normalized to the expressed housekeeping gene GAPDH. Relative expression levels were calculated with the Ct rule (Applied Biosystems, User Bulletin No. 2).

### Statistical Analysis

Each assay was performed in triplicate, and all data are expressed as mean ± SD or mean ± SEM. Statistical analyses were performed using independent samples and Student’s *t*-test or Fisher’s exact test. Two-tailed values of *P* < 0.05 were considered statistically significant. The software package SPSS version 11.0 (SPSS Inc) was used for statistical analyses.

## Results

### Genotypic and phenotypic characterization of knockout mice

**Fig 1:**
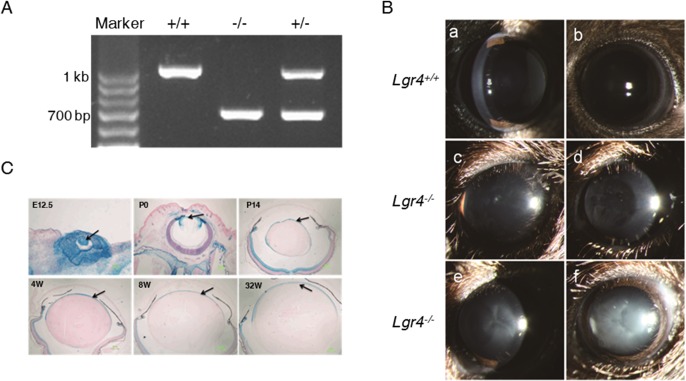
Deletion of *Lgr4* in mice causes different types of age-related cataract formation. (A) Disruption of the mouse *Lgr4* gene. Genotype analysis of *Lgr4* ^+/+^, *Lgr4* ^−/−^, and *Lgr4* ^+/−^ mice. +/+, −/−, and +/− represent genomic DNAs isolated from *Lgr4* ^+/+^, *Lgr4* ^−/−^, and *Lgr4* ^+/−^ mice, respectively. The 1 kb band is derived from the wild-type allele, and the 700 bp band is derived from the mutated allele. (B) Slit-lamp biomicroscopy examination of wild-type and *Lgr4* knockout lenses. At 20 weeks of age, diverse morphologies and various degrees of lens opacification were observed in the anterior portion of the cortex in *Lgr4* knockout mice, while most of the wild-type mice remained clear (a,b). Punctate anterior cortical cataract (c). Lamellar cortical cataract (d). The cataract region includes the Y-sutures and looks like a “fishbone” pattern (e). The whole lens becomes opaque (f). (C) Expression profile of *Lgr4* in mouse lens. LacZ staining of heterozygous lenses showed strong *Lgr4* expression in lens epithelial cells from both the embryonic and adult mice at different ages.

The presence of the mutated gene in the *Lgr4* null offspring was initially verified. As shown in Fig. 1A, PCR analysis of the null mouse genes showed that the expected recombination event had occurred. The size of the PCR product was approximately 1 kb for the wild-type allele and approximately 700 bp for the targeted allele.

Mice heterozygous for *Lgr4* and the wild-type mice had no apparent abnormalities. As we have reported [17], many homozygous *Lgr4* ^*−/−*^ mice showed ocular ASD of varying degrees (data not shown). To avoid the possibility that the onset of cataracts is a secondary result of ASD, we selected and raised mice without obvious ocular ASD and their wild-type littermates for the subsequent experiments. Slit-lamp biomicroscopy results showed that *Lgr4* ^*−/−*^ mice developed a variety of cataracts whereas most of the lenses from the wild-type mice remained clear (Fig. 1B). As lens epithelial cells play important roles in the development of cataracts, we investigated the detailed expression profile of *Lgr4* in lens epithelial cells. LacZ staining of heterozygous lenses confirmed the presence of the mutated gene in lens epithelial cells from both the embryonic and adult mice with strong staining using β-galactosidase (Fig. 1C). The expression of β-galactosidase translates into strong *Lgr4* expression assuming that the presence of the marker is a reflection of *Lgr4* expression.

### *Lgr4* deletion leads to the early onset of age-related cataract

**Fig 2:**
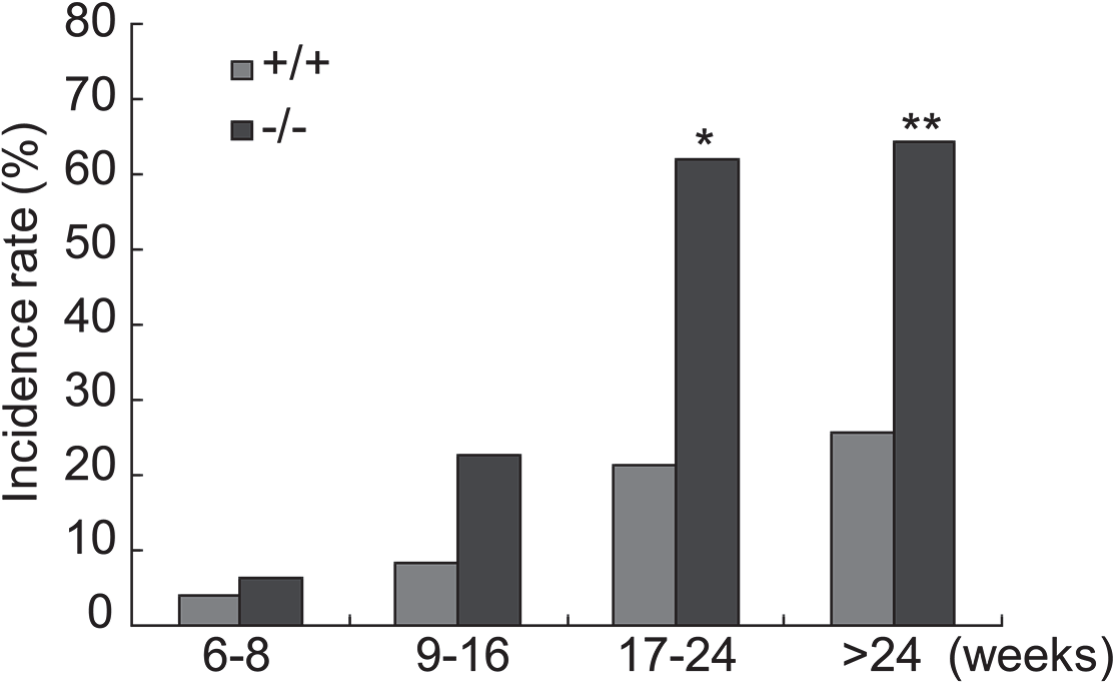
*Lgr4* deletion leads to the early onset of age-related cataract. At the age of 6 to 8 weeks (wild-type: n = 24; *Lgr4* ^−/−^: n = 16), *Lgr4* ^−/−^ mice showed no increased incidence of cataract formation. In the second stage (9 to 16 weeks, wild-type: n = 36; *Lgr4* ^−/−^: n = 22), the incidence of cataract formation was increased in *Lgr4* ^−/−^ mice. The incidence of cataracts in *Lgr4* ^−/−^ mice was significantly increased by maturity (17 to 24 weeks, wild-type: n = 42; *Lgr4* ^−/−^: n = 29) and older than 24 weeks (wild-type: n = 62; *Lgr4* ^−/−^: n = 45). *P < 0.001, **P < 0.0001.

To further analyze the cataract phenotype of *Lgr4* ^*−/−*^ mice, both direct ophthalmic examination and slit lamp microscopy were performed on lenses of homozygous *Lgr4* ^*−/−*^ and wild-type animals. A total of 112 *Lgr4* ^*−/−*^ mice and 164 wild-type mice, ranging from 6 weeks to 70 weeks, were examined. Homozygous *Lgr4* ^*−/−*^ mice with nontransparent lens accounted for about 47.3% (53/112) of total mutant adults, whereas only 17.7% (29/164) wild-type mice showed cataracts. More importantly, we were able to confirm that risk of cataract formation is progressive with increasing age well pass maturity for the mice, suggesting that *Lgr4* in cataract formation is maintained beyond the developmental stage. Many *Lgr4* knockout murine lenses showed opacification, starting at the age of 9–16 weeks (Fig. 2), with the incidence progressively worsening with advancing age. In mice older than 24 weeks, more than half of the null mice (64.4%) suffered from cataract formation in comparison to only 25.8% of the wild-type animals. These results indicated that *Lgr4* deletion leads to early onset and increased incidence of age-related cataract.

The external morphologies of lenses from the young animals (6–16 weeks) showed no obvious difference between the two groups. However, a mild loss of lens clarity with different types in mutant animals was observed after 16 weeks of age, which progressed in severity with aging. Cortical cataracts located in the anterior portion of the lenses were evident by 20 weeks of age and becomes progressive. Formation of the Y-structure along with a white nontransparent fishbone pattern occurred in the lenses by 6 months of age. In contrast, wild-type littermates were usually clear with only minor opacifications (Fig. 1B).

### *Lgr4* knockout attenuates the antioxidative ability of the lens epithelial cells

**Fig 3:**
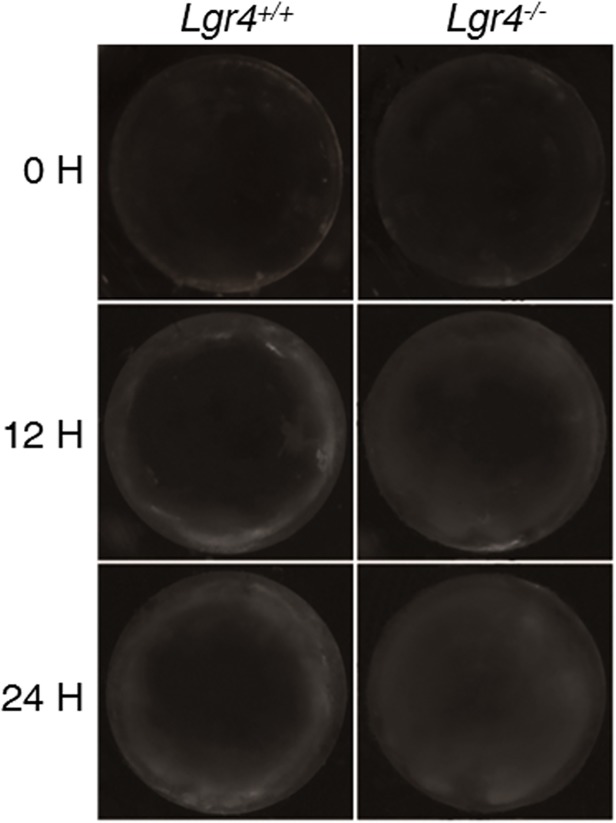
Morphologic changes of murine lenses cultured in the presence of H_2_O_2_. Lenses removed from 30-week-old mice were subjected to peroxidase damage (H_2_O_2_) and incubated for 24 hours. Lens images were captured on culture plates at 0, 12, and 24 hours after H_2_O_2_ treatment using dark field microscopy. Representative images show that the degree of lens opacity in the knockout mice was significantly more than in wild-type mice. All images are representative of at least 3 independent experiments.

To investigate the mechanisms of *Lgr4* deletion induced age-related cataract formation, we studied the ability of lens proteins to resist oxidative stress. Whole lenses from *Lgr4* ^*−/−*^ mice and their wild-type littermates at different ages were treated with 0.2 mM H_2_O_2_, and the lens opacity was observed at different times. The results showed that 10-week old animals did not display significant differences in the degree of opacity; both groups remained clear during the 24-hour procedure (data not shown). However, phenotypically normal lenses from mice older than 24 weeks (both knockout and wild-type) exposed to 0.2 mM H_2_O_2_ displayed variability in their tolerance to oxidative stress. Both lenses maintained transparency in the first 6 hours of culture. Lenses derived from *Lgr4* ^*−/−*^ mice started showing opacification at 12 hours, initially in the peripheral cortical regions. With prolonged exposure, haziness expanded to involve the entire cortex. The lenses from the *Lgr4* ^*−/−*^ mice were cloudy throughout, whereas the lenses from wild-type mice showed only slight cloudiness (Fig. 3).

**Fig 4:**
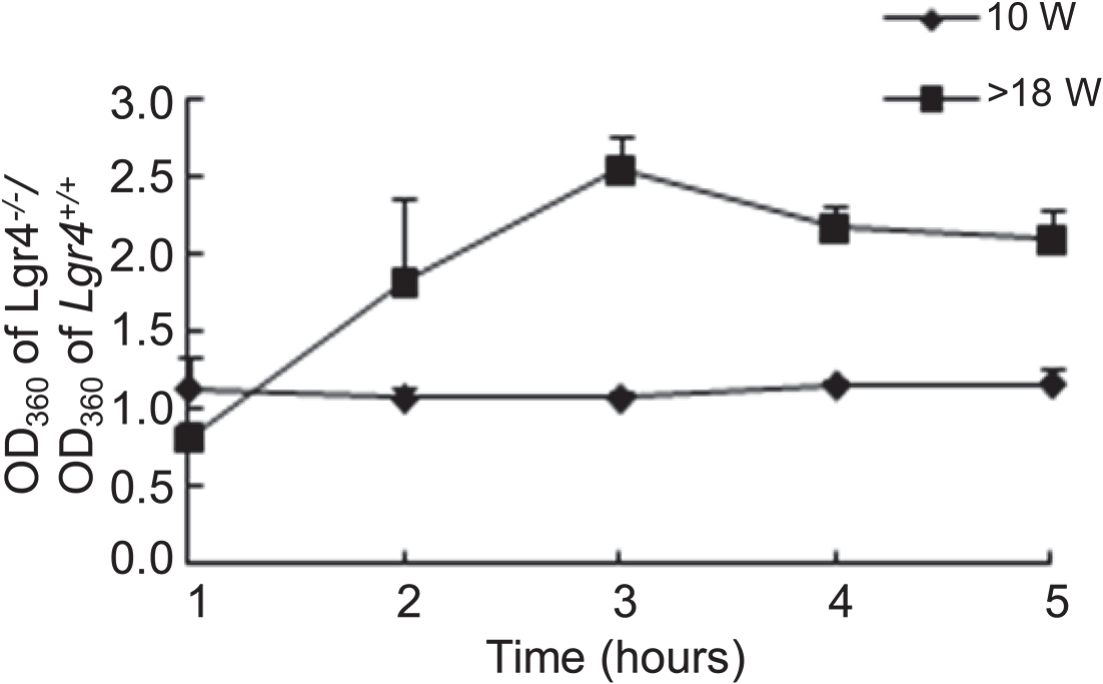
Oxidation-induced denaturation of proteins in *Lgr4* knockout lenses. Lens supernatants of 10 weeks (10 W) and >18 weeks (>18 W) diluted to 1 mg/mL protein using the TC-199 medium were subjected to oxidative damage with 0.1 mM H_2_O_2_ and 2.31 units of glucose oxidase (GO) for indicated times. Sample absorbance was measured at 360 nM. Supernatants from *Lgr4* knockout lenses showed a more rapid increase in turbidity (and therefore, protein denaturation), suggesting that the knockout lens had less overall resistance to oxidation than wild-type. Data are expressed as mean ± SD (n = 3). These results are representative of 3 independent experiments.

To further confirm that *Lgr4* deletion would attenuate the antioxidative ability of lens epithelial cells, we performed protein denaturation assay. Fresh transparent lens homogenates from *Lgr4* ^*−/−*^ mice and wild-type mice at different weeks were treated with 0.2 mM H_2_O_2_ and incubated at 37°C for indicated times. The turbidity was examined using spectrophotometry to illustrate amount of denaturation. Similar to the whole lens incubation assay, there was no significant difference between young *Lgr4* ^*−/−*^ mice and their wild-type littermates (<10 weeks). However, lens homogenates of *Lgr4* ^*−/−*^ mice denatured much faster than their comparable littermates at older age (>18 weeks) (Fig. 4). These results indicate that the deletion of *Lgr4* significantly attenuates the antioxidative ability of lens epithelial cells.

### *Lgr4* knockout down-regulates the expression of antioxidant enzymes in lens epithelial cells

**Fig 5:**
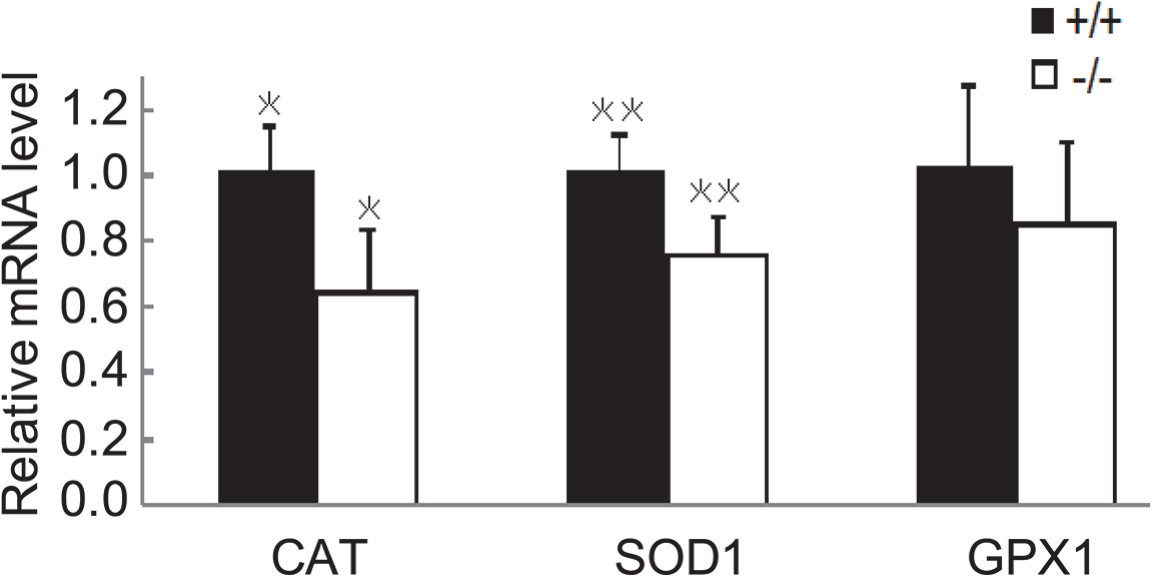
The gene expression levels of antioxidant enzymes are reduced in *Lgr4* ^−/−^ mice older than 24 weeks. The lens epithelium from *Lgr4* ^*−/−*^ mice and their wild-type littermates was isolated as described in Materials and Methods. Total RNA was extracted and the gene expression was measured by real-time PCR. The data were normalized to the level in wild-type mice. Results are expressed as mean ± SEM. n = 3, *P < 0.01.

To evaluate the expression of antioxidant enzymes in lens epithelial cells, the lens epithelium from *Lgr4* ^*−/−*^ mice and their wild-type littermates of 24 weeks old was removed as described. Total RNA was extracted and the gene expression was measured by real-time PCR. Of the three antioxidant defense enzymes, the expressions of catalase (CAT) and superoxidase dismutase-1 (SOD1) were significantly down-regulated in *Lgr4* ^*−/−*^ mice compared to those in wild-type animals. The gene glutathione peroxidase-1 (GPX1) was not changed under the present experimental conditions (Fig. 5).

## Discussion

LGR4 has been implicated in many physiological processes including embryonic development, cell motility, and tumor metastasis [10–15,20–27]. Recently, a nonsense mutation (c.376C>T) of *LGR4*, which abolishes the signal transduction of LGR4, has been reported in humans to be strongly associated with low bone mineral density, osteoporotic features, electrolyte imbalances, reduced testosterone levels, and increased risk of cutaneous squamous cell cancers and biliary tract cancers [28]. Interestingly, these characteristics overlap with those of *Lgr4* knockout mice [28]. This supports the notion that LGR4 may be important in human development as well. The role of LGR4 in mice has been well established; yet, its roles *in vivo* under normal physiological conditions remain poorly understood with regards to the pathogenesis of lens opacification.

In this study, we sought to investigate the role of *Lgr4* in cataract formation using a line of *Lgr4* knockout mice. We found *Lgr4* was strongly expressed in lens epithelial cells from both the embryonic and adult mice. We selected and raised *Lgr4* ^*−/−*^ mice without obvious ASD and their wild-type littermates for experiments to avoid the possibility that the onset of cataract is a secondary result of ASD. We found that *Lgr4* ^*−/−*^ mice established early onset cataract, and the lens opacification was progressive in severity. More importantly, the incidence of cataract formation was much higher in *Lgr4* ^*−/−*^ mice compared with wild-type of the same age. These results indicated that *Lgr4* mitigates the onset of age-related cataract formation.

To elucidate the mechanisms of *Lgr4* mediated age-related cataract formation, the lens from *Lgr4* knockout mice was treated with H_2_O_2_, and the resultant lens opacification was imaged. Moreover, we also tested the stability of the total lens protein, most of which are crystallins, in younger and older *Lgr4* ^*−/−*^ mice. All the results indicated that *Lgr4* deletion attenuates the antioxidant capacity of the lens.

We performed early initial investigations into the cause of *Lgr4* related cataract formation with analysis of the lens gap junctions. Lens epithelial and fiber cells contain distinct gap junctions, oligomeric proteins called connexins (Cx) [29]. There are three connexins identified in the lens: Cx43 is expressed only in epithelial cells, Cx46 is expressed only in fiber cells, and Cx50 is expressed in both epithelial cells and fiber cells [30]. Using Western blot analysis and immunofluorescence assay, no marked defects of the three connexins were observed in the lenses of *Lgr4* knockout compared with wild-type (data not shown). These results suggest that knockout of *Lgr4* gene does not increase cataract formation through disruption of the gap junction channels.

One of the mechanisms by which mammals prevent age-related cataract formation is up-regulation of antioxidant defense enzymes, which are mainly expressed by the lens epithelial cells. We isolated the lens epithelium from *Lgr4* ^*−/−*^ mice and their wild-type littermates, and the expression of three main antioxidant defense enzymes (CAT, SOD1, GPX1) was evaluated by real-time quantitative PCR. The results showed that CAT and SOD1 were significantly reduced due to *Lgr4* deletion. Reactive oxygen species (ROS) including the superoxide anion (O_2_^-^), hydroxyl free radicals (OH^-^), and H_2_O_2_ may interfere with the redox state of the lens, and contribute to cataract formation [31]. SOD1 catalyzes the conversion of O_2_^-^ to H_2_O_2_. H_2_O_2_ can be degraded enzymatically by CAT, an enzyme that specifically uses H_2_O_2_ as a substrate, or by GPX1, a selenoenzyme that reduces peroxides and requires glutathione [32]. Thus, our data indicate that *Lgr4* deletion may accelerate the onset of age-related cataract formation through down-regulation of SOD1 and CAT.

The process of LGR4 regulation of the antioxidant enzymes in lens epithelial cells remains elusive. Several pathways have been suggested for LGR4 regulation of antioxidant enzymes. LGR4 has long been considered an orphan receptor until recent discoveries identified R-spondin as the extracellular ligand of LGR4 [33–35]. R-spondin binding to LGR4 potentiates Wnt signaling pathway by inhibiting ZNR3 and RNF43, which promotes degradation of the Wnt receptor Frz and LRP5/6 [36]. LGR4 mediated Wnt signaling pathway has been implicated in stem cell maintenance in many tissues [34]. In addition, LGR4 has been shown to signal through classical G protein-coupled receptor signaling in multiple tissues. In this pathway, cyclin AMP (cAMP) is activated due to activation of LGR4, and cAMP activates protein kinase A (PKA) which in turn phosphorylates the transcription factor Cre-binding protein leading to expression of target genes. Reported LGR4 targets regulated by cAMP/PKA pathway include the estrogen receptor α, ATF4, Pitx2, and mineralocorticoids [37]. However, the ligands that activate cAMP/PKA signaling pathway remain unidentified. Our group has previously reported that LGR4 regulates the proliferation and migration of keratinocyte through HB-EGF mediated EGFR transactivation [15]. Wnt, cAMP/PKA and EGFR signaling pathways have all been reported to be involved in the regulation of antioxidative stress ability in lens epithelial cells [38,39]. However, the pathway responsible for LGR4 mediated development of age-related cataract still needs further investigation.

In conclusion, *Lgr4* knockout mice developed age-related cataract at 17 to 24 weeks after birth. The opacification begins in the anterior and peripheral cortical zone and the nucleus of the lens, and progresses both in extent and severity with increasing age. The lens proteins showed reduced resistance to oxidative stress. Expression levels of CAT and SOD1 are decreased in *Lgr4* ^*−/−*^ lens. These results suggest murine knockout of *Lgr4* gene induces age-related cataract by down-regulating the gene expression of antioxidant defense enzymes in lens epithelial cells.
